# Whole-heart contrast-enhanced coronary magnetic resonance angiography in less than 5 minutes using gradient echo interleaved EPI

**DOI:** 10.1186/1532-429X-11-S1-O47

**Published:** 2009-01-28

**Authors:** Himanshu Bhat, Sven Zuehlsdorff, Xiaoming Bi, Xin Liu, Renate Jerecic, Debiao Li

**Affiliations:** 1 Northwestern University, Chicago, IL USA; 2 Siemens Medical Solutions, Chicago, IL USA

**Keywords:** Imaging Time, Image Ghost, Coronary Magnetic Resonance Angiography, Coronary Artery Image, High Acceleration Factor

## Introduction

Whole-heart coronary MRA is challenging due to the relatively long data acquisition time on the order of 10–15 minutes [1]. Interleaved EPI [2] is a method which can be exploited to provide significant speed gain for whole-heart coronary MRA and has previously been reported for volume-targeted imaging at 1.5 T [3, 4]. The purpose of this work was to optimize an interleaved EPI acquisition scheme for reducing the imaging time of whole-heart contrast-enhanced coronary MRA.

## Methods

### Sequence design considerations

A schematic of the EPI-FLASH sequence is shown in Fig. 1a. Segmentation and interleaving were applied along the phase-encoding direction, eliminating discrete signal variations in the partition-encoding direction. The reordering scheme used in the phase-encoding direction is shown in Fig. 1b. The asymmetric k-space was divided into 6 regions corresponding to the echo train length, using the second region as central k-space region. The interleaved acquisition within a heartbeat initially samples the lower portion of each region (dashed line) and subsequently sequentially acquires the central k-space line and top of each region (dotted line). The signal varies within each heartbeat due to non steady-state conditions. In combination with the described reordering scheme, this results in amplitude modulations in k-space, leading to image ghosts [2]. These modulations were minimized by appropriate selection of the inversion time and flip angle, using simulations of the Bloch equations and phantom studies.

**Figure:**
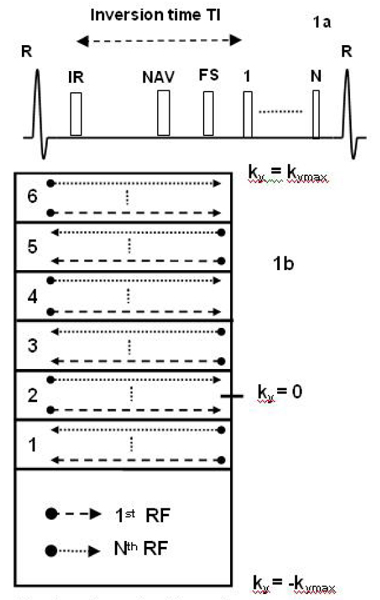
Figure 1 **(a) schematic of the pulse sequence; (b) reordering scheme**.

### Volunteer imaging

7 volunteers were scanned on a 1.5 T Espree scanner (Siemens Medical Solutions). Scan parameters were: TR = 11.3, TE = 4.27, flip angle = 25, 66 lines per heartbeat in a window of 124 ms, acquired k-space lines = 132, readout bandwidth = 977 Hz/pixel, TI = 300 ms, matrix: 256 × 189 × 60, interpolated voxel size: 0.5 × 0.55 × 1 mm^3^. 0.2 mmol/kg body weight of Gd-DTPA was injected at 0.5 cc/sec [5]. The total imaging time for the whole-heart scan was 2 minutes (for a heart-rate of 60 without navigator gating). For comparison, a 3D TrueFISP whole-heart scan was acquired using matched data acquisition time and spatial resolution. The image quality scores (1, poor; 2, fair; 3, good; 4, excellent) and lengths of the coronary arteries visualized by the 2 techniques were compared. In addition, for a qualitative comparison, a whole-heart TrueFISP protocol [5] with longer scan time, representing the state of the art for coronary MRA at 1.5 T was performed on 2 of the volunteers.

## Results

**Figure 2:**
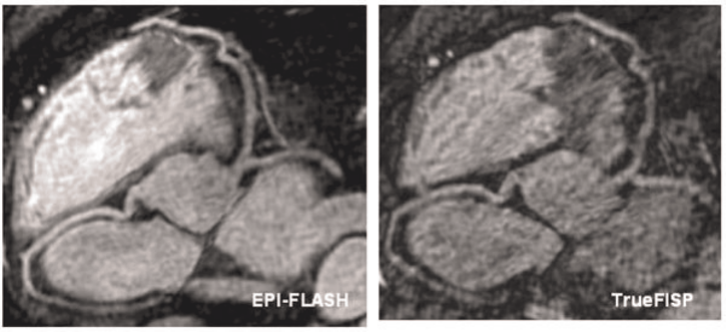
**Coronary artery images using the EPI-FLASH and TrueFISP sequences with identical imaging times**.

**Figure 3:**
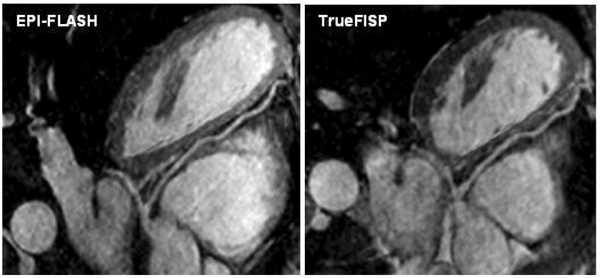
**Coronary artery images using the EPI-FLASH sequence and a TrueFISP sequence with longer imaging time**.

The average imaging time for contrast-enhanced whole-heart imaging was 4.7 ± 0.7 minutes with an average navigator efficiency of 44.7 ± 6.2%. Fig. 2 shows coronary artery images from 2 volunteers using the EPI-FLASH acquisition and the TrueFISP acquisition with identical imaging time. The EPI-FLASH acquisition shows excellent depiction of all the coronary arteries. In comparison, the TrueFISP acquisition is very noise due to the high acceleration factor used. Quantitative comparison between the two sequences is shown in Table 1. Fig. 3 shows coronary artery images from a volunteer using the EPI-FLASH acquisition (imaging time = 5.6 minutes, navigator efficiency = 45%) and a TrueFISP acquisition (imaging time = 13.2 minutes, navigator efficiency = 38%). Both the sequences show similar depiction of the coronary arteries, but the imaging time for the EPI-FLASH technique is reduced by more than a factor of 2.

**Table 1:** Comparison between EPI-FLASH and TrueFISP with identical resolution and imaging time

Sequence	Imaging time	Navigator efficiency	Image quality score	RCA length	LAD length
EPI-FLASH	4.7 ± 0.7	44.7 ± 6.2	3 ± 0.3	10.8 ± 2.1	11.5 ± 3.2
TrueFISP	4.9 ± 0.9	45.5 ± 7.9	2.2 ± 0.3	9.1 ± 1.72	11.0 ± 4.1
p value (n = 7)	0.5	0.8	0.001*	0.01*	0.4

## Conclusion

An EPI-FLASH sequence was optimized for contrast-enhanced whole-heart coronary MRA at 1.5 T. In volunteers, all the major coronary arteries were clearly depicted in a scan time under 5 minutes. Clinical utility of the technique needs to be tested on a patient population.
